# Influence of the First Wave of the COVID-19 Pandemic on Cancer Care in a German Comprehensive Cancer Center

**DOI:** 10.3389/fpubh.2021.750479

**Published:** 2021-11-23

**Authors:** Johanna Kirchberg, Anke Rentsch, Anna Klimova, Vasyl Vovk, Sebastian Hempel, Gunnar Folprecht, Mechthild Krause, Verena Plodeck, Thilo Welsch, Jürgen Weitz, Johannes Fritzmann

**Affiliations:** ^1^Department of Visceral, Thoracic and Vascular Surgery, University Hospital Carl Gustav Carus, Technische Universität Dresden, Dresden, Germany; ^2^National Center for Tumor Diseases (NCT/UCC), Dresden, Germany; German Cancer Research Center (DKFZ), Heidelberg, Germany; Faculty of Medicine and University Hospital Carl Gustav Carus, Technische Universität Dresden, Dresden, Germany; Helmholtz-Zentrum Dresden - Rossendorf (HZDR), Dresden, Germany; ^3^Department of Radiation Oncology, Faculty of Medicine and University Hospital Carl Gustav Carus, Technische Universität Dresden, Dresden, Germany; ^4^German Cancer Research Center (DKFZ), German Cancer Consortium (DKTK), Partner Site Dresden, Heidelberg, Germany; ^5^OncoRay - National Center for Radiation Research in Oncology, Faculty of Medicine and University Hospital Carl Gustav Carus, Technische Universität Dresden, Helmholtz-Zentrum Dresden - Rossendorf, Dresden, Germany; ^6^Helmholtz-Zentrum Dresden - Rossendorf, Dresden, Germany; ^7^Department of Diagnostic and Interventional Radiology, University Hospital Carl Gustav Carus, Technische Universität Dresden, Dresden, Germany

**Keywords:** COVID-19 pandemic, cancer care, German, health care, comprehensive cancer center

## Abstract

**Introduction:** During the first wave of the COVID-19 pandemic in 2020, the German government implemented legal restrictions to avoid the overloading of intensive care units by patients with COVID-19. The influence of these effects on diagnosis and treatment of cancer in Germany is largely unknown.

**Methods:** To evaluate the effect of the first wave of the COVID-19 pandemic on tumor board presentations in a high-volume tertiary referral center (the German Comprehensive Cancer Center NCT/UCC Dresden), we compared the number of presentations of gastrointestinal tumors stratified by tumor entity, tumor stage, and treatment intention during the pandemic to the respective data from previous years.

**Results:** The number of presentations decreased by 3.2% (95% CI −8.8, 2.7) during the COVID year 2020 compared with the pre-COVID year 2019. During the first shutdown, March–May 2020, the total number of presentations was 9.4% (−18.7, 1) less than during March–May 2019. This decrease was significant for curable cases of esophageal cancer [*N* = 37, 25.5% (−41.8, −4.4)] and colon cancer [*N* = 36, 17.5% (−32.6, 1.1)] as well as for all cases of biliary tract cancer [*N* = 26, 50% (−69.9, −15)] during the first shutdown from March 2020 to May 2020.

**Conclusion:** The impact of the COVID-19 pandemic on the presentation of oncological patients in a CCC in Germany was considerable and should be taken into account when making decisions regarding future pandemics.

## Key Points

- The impact of the COVID-19 pandemic on the presentation of oncological patients in a CCC in Germany was considerable.
- The number of presentations decreased by 3.2% during the COVID-year 2020 compared with the pre-COVID year 2019 and more pronounced by 9.4% during the first shutdown from March 2020 to May 2020 compared with March 2019 to May 2019.
- This decrease was significant for curable cases of esophageal cancer and colon cancer as well as biliary tract cancer during the first shutdown from March 2020 to May 2020.
- Targeted countermeasures should be taken to avoid disastrous effects on oncological patients, as the pandemic is still ongoing.

## Introduction

The COVID-19 pandemic posed unprecedented challenges to healthcare systems worldwide. On the one hand, the treatment of patients with COVID-19 competed with cancer treatment due to limited hospital resources. On the other hand, patients showed fear-related avoidance behavior and delayed clinical visits for symptoms or newly diagnosed diseases. The main challenge was to create resources for the treatment of patients with COVID-19 while maintaining the treatment of other severe diseases.

Due to the high number of patients with COVID-19 in Germany in March 2020, a national hospital relief law came into effect in April 2020, which served to keep hospital capacities free for patients with COVID-19 (1). The federal government, thus, imposed the first shutdown of public life on March 27, 2020, which was largely lifted on May 6, 2020 (2). Furthermore, outpatient and inpatient health facilities adapted the levels of care to manage the local pandemic situation (3).

In general, the first COVID-19 wave in Germany took place in 2020 with a peak from March 2020 to April 2020. The first hard shutdown in Germany was imposed from March 2020 to May 2020. From May 2020 to September 2020, the pandemic situation calmed down. The second wave took place from October 2020 to January 2021 (4).

According to a survey of 18 German comprehensive cancer centers (CCCs) by the German Cancer Society, tumor follow-up examinations were limited to 60–80% during the first 6 weeks of the pandemic from the end of March to the beginning of May.

At NCT/UCC, analogous to the first national shutdown, nonurgent tumor follow-up examinations were postponed or performed by phone or video conferences with patients with gastrointestinal tumors to protect patients from the risk of virus exposure. Chemotherapy, radiotherapy, and tumor operations were performed largely normally during the first shutdown. Daily tumor board presentations were continued as online video boards with unchanged staff lineup and frequency.

Significant negative effects resulting from these adjustments to emergency patient and tumor patient care have been reported (3, 5–8). There was also a considerable decrease in the number of newly diagnosed cancer cases (9–13).

Nevertheless, the long-term consequences of the abovementioned healthcare adjustments during the COVID-19 pandemic for cancer patients are largely unclear (3, 14). A stage shift toward higher tumor stages at initial cancer diagnosis is feared in future years (14, 15).

Hanna et al. were able to show in a meta-analysis from the pre-COVID-19 era that even a 4-week delay of cancer treatment is associated with increased mortality across surgical, systemic, and radiotherapy treatments for seven cancer types (16).

The need for an improved understanding of the impact of treatment delay on outcomes has come into focus during the COVID-19 pandemic and represented the rationale for carrying out this study.

Therefore, we aimed to reveal the influence of the first wave of the COVID-19 pandemic (January 2020–October 2020) and during the first national shutdown (March 2020–May 2020) on the care of oncological patients at the CCC NCT/UCC Dresden compared with the pre-COVID-19 era (2014–2019).

## Methods

The frequency of tumor board presentations of gastrointestinal tumors at the NCT/UCC Dresden before (2014–2019) and during the first wave of the COVID-19 pandemic (January 2020–October 2020), and during the first national shutdown (May 2020–May 2020) was analyzed retrospectively.

All cases of malignant gastrointestinal tumor entities [biliary tract (gall bladder, extrahepatic bile duct, ampulla vateri); esophagus; colon; rectum; pancreas; liver (hepatocellular carcinoma, HCC, and intrahepatic bile duct); stomach (including esophagogastric junction); others] that were presented to our tumor board during the specific time period were included in the analysis.

A multidisciplinary treatment plan during the tumor board was designed by five independent experts (surgical oncologist, medical oncologist, radiation therapist, radiologist, and pathologist) who regularly participated in the interdisciplinary tumor boards. These experts also defined treatment intention (curative, palliative, or not yet decided).

The data were extracted from our institutional tumor documentation system. Data collection and statistical analysis of the number of tumor board presentations from 2014 to October 2020 was carried out by in-house mathematicians/statisticians.

Each relative percentage change from one period to another was calculated based on the observed total counts over the corresponding two time periods and was shown with a 95% confidence interval. The intervals were computed under the assumption that monthly counts follow a Poisson distribution, using the shrunk logit Wald method (17). Time series analyses were implemented using the R package tscount (18).

A statistical analysis of the data was carried out with SPSS Version 27 (IBM, Armonk, NY, United States), and a time series analysis was performed with the R environment for statistical computing (19). A statistical analysis plan was created before undertaking the analyses.

At the first treatment at the NCT/UCC, all the patients consented to the anonymous scientific evaluation of their data and signed a written consent form, which was approved in advance by the local ethics committee.

## Results

### Baseline Characteristics

A total of 15,995 tumor board presentations of gastrointestinal tumors in 7,263 patients took place across 82 months from 2014 to October 2020 (Table 1).

**Table 1:** Baseline characteristics of tumor board presentations in the comprehensive cancer center (CCC) from January 2014 to October 2020.

	***N* (%)**
**Number of tumor board presentations**	15,995
**Age (median, range)**	66,08 (17.9–97.6)
**Sex**	
M	10,613 (66.4)
W	5,382 (33.6)
**Reason for presentation**	
Primary tumor	7,575 (47.4)
Local recurrence	1,190 (7.4)
Metastases	7,218 (45.1)
Others	12 (0.1)
**Intention of therapy**	
Curative	8,546 (52.9)
Palliative	4,883 (30.5)
Not yet decided	2,460 (15.4)
Not applicable	196 (1.2)
**Death by last follow-up**	
Yes	5,947 (37.2)
No	10,048 (62.8)

The median patient age at presentation was 66 years; 66.4% of the patients were male, and 33.6% of the patients were female.

The most common tumor entities that led to tumor board presentation were rectal cancer (15.7%), followed by colon cancer (14.3%), pancreatic cancer (13.1%), and liver cancer (11.6%) (HCC and intrahepatic bile duct cancer) (Supplementary Table S1).

### Development of Case Numbers at NCT/UCC Before the COVID-19 Pandemic

Before the COVID-19 pandemic, from 2014 to 2019, there was continuous growth in the number of tumor board presentations per year (Table 2). The percentage change in tumor board presentations per year ranged from a single decrease of 0.9% (−6.5, 5.1) in 2016 to 2017 to a maximal increase of 14.4% (8.1, 21) in 2017 to 2018, with a total average yearly increase of 6% (3.2, 8.5).

**Table 2:** The number of tumor board presentations per year from 2014 to 2020 and change compared with the previous year.

	**Total/year (*N*)**	**Change/year**
		***N* [% of year before (95 % CI)]**
2014	1,928	–
2015	2,116	+ 188 [+ 9.8% (3.2, 16.7)]
2016	2,282	+ 166 [+ 7.8% (1.6, 14.4)]
2017	2,262	– 20 [− 0.9% (−6.5, 5.1)]
2018	2,587	+ 325 [+ 14.4% (8.1, 21.0)]
2019	2,659	+ 72 [+ 2.8% (−2.6, 8.5)]
01/2020–10/2020	2,161	–
Total	15,995	+ 731 [+ 6% (3.2, 8.5)]^*^

* *Average increase/year, time series analysis with a linear trend 2014–2019*.

### Development of Case Numbers at NCT/UCC During the COVID-19 Pandemic

Before the COVID-19 pandemic, from January 2019 to October 2019, there were 2,233 tumor board presentations, and during the COVID-19 pandemic, from January 2020 to October 2020, there were 2,161 tumor board presentations. This equals a decrease of 3.2% (−8.8, 2.7) in 2020 compared with 2019.

In the time series analysis, based on the total number of tumor board presentation rates from 2014 to 2019, we found that the predicted observations for the whole year of 2020 were within the prediction intervals (data not shown).

**Figure 1:**
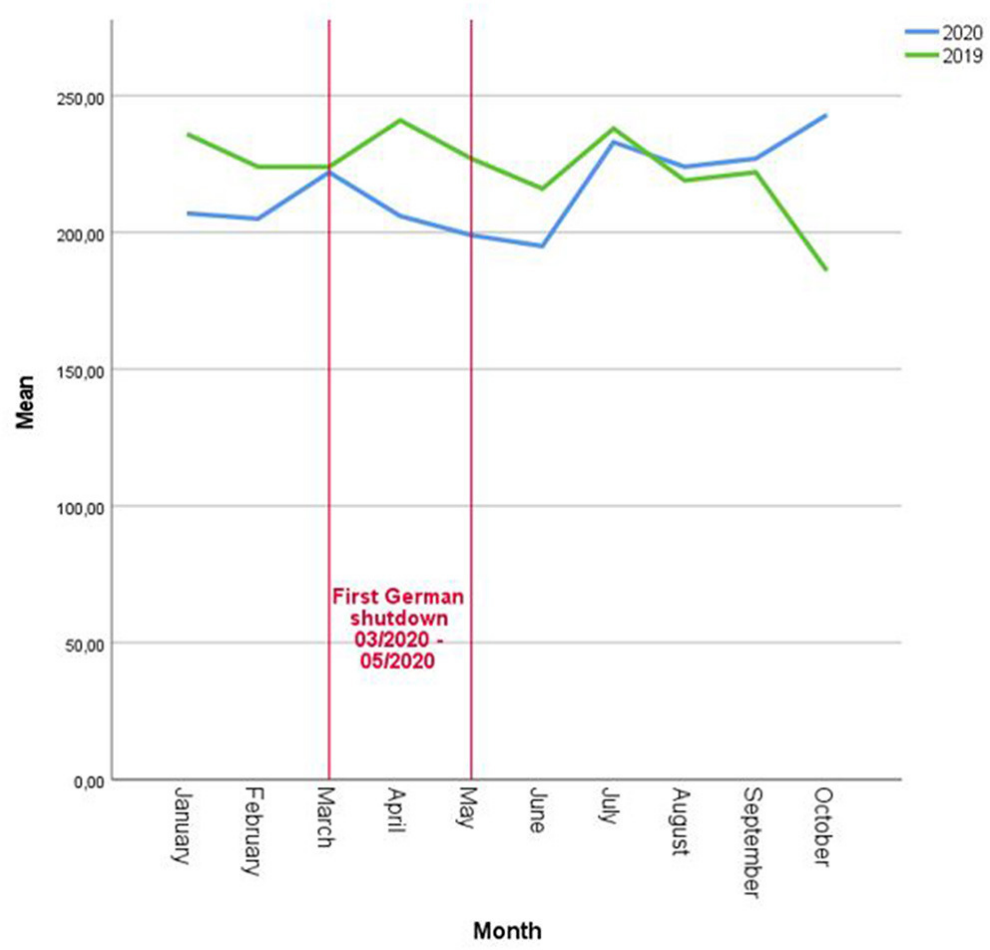
The number of tumor board presentations per month/year 2019 (pre-COVID-19 pandemic) vs. 2020 (during COVID-19 pandemic).

Compared with the equal time span in 2019, the number of presented cases decreased after the beginning of the first shutdown in March 2020 in Germany (March 2020–May 2020) and continued for at least 5 months until July 2020 (Figure 1).

During the first shutdown, March 2020-May 2020, the total number of presentations was 9.4% (−18.7, 1) less than during the equal time span 2019.

### Development of Case Numbers at NCT/UCC Based on Tumor Entity

From 2014 to 2020, there was an average annual increase in the presentation rate across all tumor entities from 2.9%/year (0.1, 5.8) for gastric cancer to 10%/year (5.7, 14.6) for pancreatic malignancy (Table 3).

**Table 3:** The average change in tumor board presentation rate per tumor entity and year from 2014 to 2020, January 2019 to October 2019 (pre-COVID-19 pandemic) vs. January 2020 to October 2020 (during COVID-19 pandemic) and during the shutdown period from March 2020 to May 2020 compared with March 2019 to May 2019.

**Tumor entity**	**Average change/year**	**01-10/2019**	**01-10/2020**	**Change 2020 vs. 2019**	**Average change from**
	**2014–2020**				**March to May 2020**
	[%, (95% CI)]^*^				vs. 2019^*^
		***N***	***N***	***N* [% (95% CI)]**	***N* [% (95% CI)]**
Biliary tract (gall bladder, extrahepatic bile duct, ampulla vateri)	+8.1% (1.3, 15.3)	110	92	−18 [−16.4% (−36.5, 10.3)]	−26 [−50% (−69.9, −15.0)]
Esophagus	+4.5% (1.0, 8.0)	220	194	−26 [−11.8% (−27.3, 7.0)]	−10 [−15.2% (−40.4, 21.1)]
Colon	+5.1% (1.3, 9.0)	337	305	−32 [−9.5% (−22.5, 5.7)]	−5 [−5.1% (−28.5, 26.0)]
Pancreas	+10% (5.7, 14.6)	327	300	−27 [−8.3% (−21.5, 7.3)]	−29 [−17.6% (−37.6, 9.1)]
Rectum	+4.1% (1.2, 7.1)	374	358	−16 [−4.3% (−17.2, 10.6)]	−4 [−3.9% (−27.0, 26.6)]
Liver (HCC and intrahepatic bile duct)	+6.0% (1.7, 10.4)	247	242	−5 [−2.0% (−17.9, 17.0)]	−12 [−13.5% (−32.6, 11.0)]
Stomach (incl. esophagogastric junction)	+2.9% (0.1, 5.8)	211	247	+36 [+17.0% (−2.6, 40.6)]	+6 [+8.8% (−21.7, 51.0)]

* *Time series analysis with a linear trend*.

There was a decrease in the frequency of tumor board presentations across all gastrointestinal tumor entities at our center during the COVID-19 pandemic in 2020 compared with 2019, except for gastric cancer.

A maximal reduction in the presentation rate of 16.4 (−36.5, 10.3) and 11.8% (−27.3, 7) is found for biliary tract and esophageal cancers, respectively, followed by colon cancer [9.5% (−22.5, 5.7)], pancreatic cancer [8.3% (−21.5, 7.3)], and rectal cancer [4.3% (−17.2, 10.6)].

The targeted comparison of the shutdown period from March 2020 to May 2020 with the same time period 2019 only showed a significant decrease of 50% (−69.9, −15) for tumors of the biliary tract independently of tumor stage or treatment intention.

**Figure 2:**
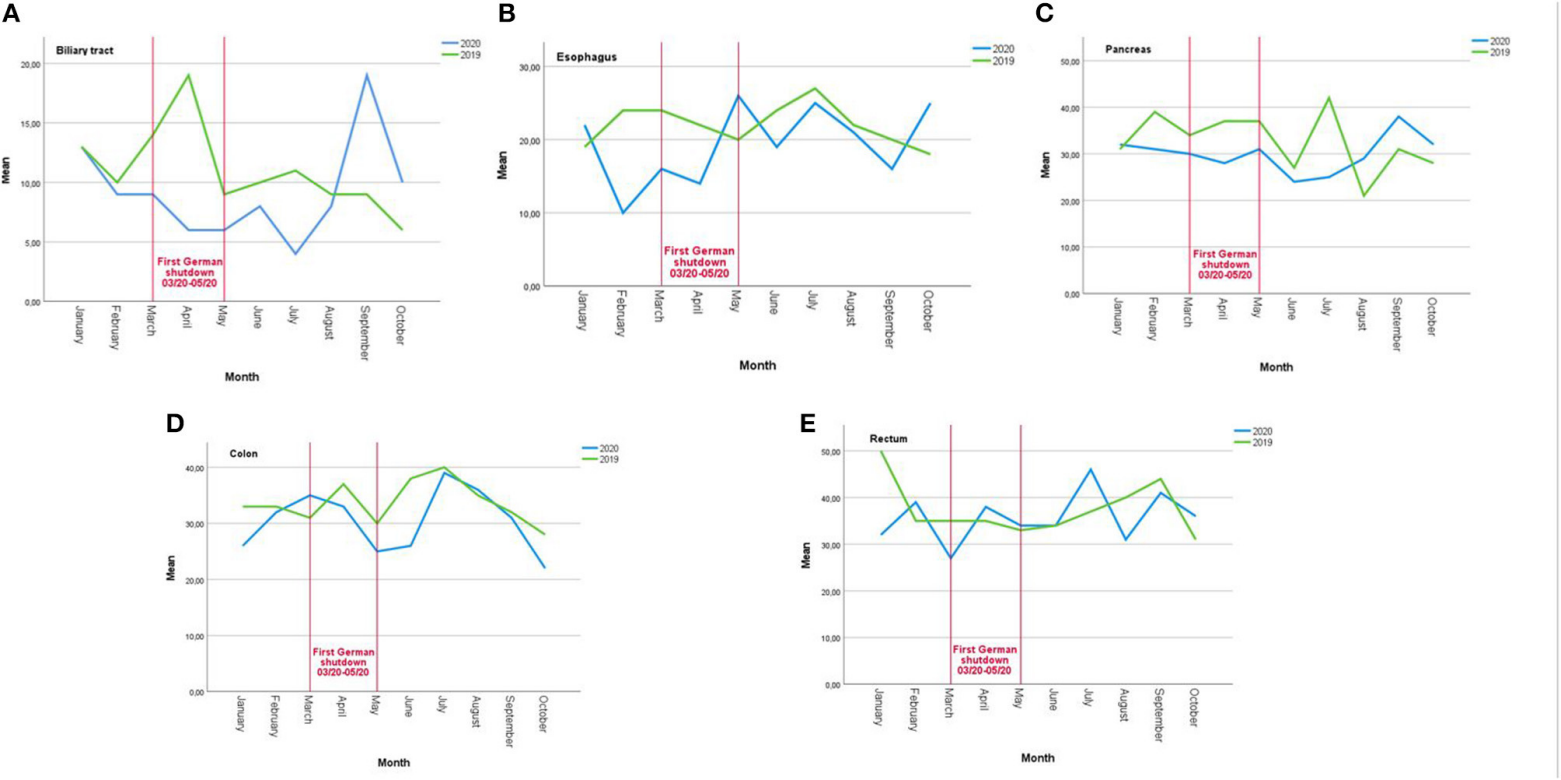
Number of tumor board presentations for different cancer entities per month/year 2019 (pre-COVID-19 pandemic) vs. 2020 (during COVID-19 pandemic); **(A)** biliary tract, **(B)** esophagus, **(C)** pancreas, **(D)** colon, and **(E)** rectum.

The rates of tumor board presentations stratified by tumor entity are depicted in Figure 2.

### Development of Case Numbers at NCT/UCC Based on Treatment Intention (Curative vs. Palliative vs. Not Yet Decided)

From 2014 to 2020, there was a considerable average annual increase of 3.9% (1.7, 6.1) in curative and 4.4% (2.2, 6.7) in palliative presentations.

In both 2019 and 2020, ~50% of the presentations concerned curative intentions and, ~30% discussed palliative treatment intentions (Supplementary Table S2).

Remarkably, there was a borderline significant decrease of 7.1% (−14.4, 0.9) in the presentation of curative intentions throughout 2020 during the COVID-19 pandemic. A similar decrease was not identified in presentations regarding palliative treatment intentions [+0.2% (−10.1, 11.6)].

During the shutdown period from March 2020 to May 2020, there was also a remarkable but not yet significant decrease in local recurrent cases of 20.8% (−46.9, 18.8) compared with the same period in the previous year.

In 2020, the number of patients with esophageal cancer who presented with potentially curable disease decreased significantly by 37% compared with the number in 2019, corresponding to a relative change of −25.5% (−41.8, −4.4) (Supplementary Table S3).

There was also a borderline significant decrease in the number of curable cases of colon cancer in 2020 during the COVID-19 pandemic compared with 2019 by 17.5% (−32.6, 1.1).

### Development of Case Numbers at NCT/UCC Based on Tumor Stage (Primary Tumor vs. Metastases vs. Local Recurrence)

From 2014 to 2020, the rate of tumor board presentations of primary tumors, local recurrent tumors, and metastases constantly increased, with an average yearly increase of 6.2 (2.6, 10.1), 10.4 (3.7, 17.5), and 4.7% (1.5, 8.1), respectively. This corresponds to the general trend of treating increasingly more complex cases at the center over time (Supplementary Table S4).

During the COVID-19 pandemic in 2020, compared with 2019, there was a decrease in the tumor board presentation rate for all tumor stages, which was most pronounced for locally recurrent tumors [7.6% (−25.5, 14.7)].

When the tumor presentation was stratified by tumor type, we observed a remarkable but not significant decrease in primary tumors, especially in esophageal cancer [13.7% (−33.8, 12.7)], colon cancer [11.8% (−32.8, 15.9)], and rectal cancer [12% (−32.4, 14.7)] in 2020 compared with 2019 (Supplementary Table S5).

During the shutdown period from March 2020 to May 2020, there was also a remarkable but not yet significant decrease in local recurrent cases of 20.8% (−46.9, 18.8) compared with the same period in the previous year.

## Discussion

The 2020 COVID-19 pandemic affected healthcare systems worldwide and brought unprecedented challenges. Patient and physician behavior following the political decision to implement a shutdown had serious consequences on the diagnosis, treatment, and outcome of severe diseases, such as cancer (3, 6). The analysis of our data showed a decrease in the number of tumor board presentations during the 2020 COVID-19 pandemic compared with the pre-COVID-19 era for all gastrointestinal tumor entities.

This decrease was significant in the COVID-19-year 2020 for curable cases of esophageal cancer [13.7% (−33.8, 12.7)] and curable cases of colon cancer [−17.5% (−32.6, 1.1)] as well as for all cases of biliary tract cancer [50% (−69.9, −15)] during the first shutdown period from March 2020 to May 2020.

To the best of our knowledge, our study is the first to differentiate and analyze the influence of the COVID-19 pandemic on gastrointestinal carcinomas stratified by type. Other available publications typically discuss either gastrointestinal tumors without subtyping (9, 11), colorectal carcinoma (10, 13, 20), or endoscopically diagnosable cancers (12) alone.

Morris, Turnbull, and Dinmohamed (9, 10, 13, 20) showed based on national registry data from the United Kingdom and the Netherlands that there was a sustained reduction in the number of colorectal cancer [22% (13, 20)] and all cancer sites [26% (9, 10)] referred for treatment during the first wave of the COVID-19 pandemic. Dinmohamed et al. did not present a sub-analysis within the group of gastrointestinal cancers.

The findings of this study emphasize for the first time a demonstrably negative effect of the first wave of the COVID-19 pandemic on the presentation of biliary tract cancer, esophageal cancer, and colon cancer in a German CCC.

As esophageal and colorectal cancers are usually diagnosed endoscopically, a well-known reduction in endoscopies (12) during the pandemic might explain the significant reduction in the number of curable cases of these entities presented at our center during the first wave of the COVID-19 pandemic.

The data of Rutter et al. strongly support this (12). A weekly average of 35,478 endoscopy procedures was performed in the pre-COVID-19 period in the UK. Activity in the COVID-19 period reduced to 12% of the pre-COVID-19 levels. The proportion of the decrease in the numbers of cancers diagnosed ranged from 19% (pancreatobiliary) to 37% (esophageal) to 72% (colorectal) (12).

Of course, there are also regional influencing factors. As a university hospital, our center focuses on extended oncologic procedures, such as major liver and robotic esophageal surgery as well as advanced multivisceral resections of recurrent cancers and receives many external referrals from supraregional hospitals for these tumors. This might have influenced our data because of the reduced mobility of patients during the pandemic.

Whether the delay in making the diagnosis or the delay in referral to the center will have a negative impact on patient survival can only be speculated. Nevertheless, it seems likely, as this correlation has been described in numerous publications (15, 21–23). The so-called stage shift caused by the COVID-19 pandemic can only be reliably analyzed several years after the pandemic (3, 24, 25).

To our knowledge, the rate of tumor board presentations as a surrogate marker for pandemic-related impact on outpatient and inpatient oncological care has not been investigated yet. In our opinion, there are two arguments for the validity of the tumor board presentation rate parameter.

On the one hand, the most frequently affected tumor entities in our center are similar to those described in other publications based on registry data (9, 11, 13).

On the other hand, the decrease in presentation rate over time coincides with the beginning of the first shutdown in Germany. After the end of the first shutdown in May 2020, rates recovered after a delay of a few months. Piontek et al. recently analyzed cancer incidence rates of the clinical cancer registry in Saxony during the pandemic between January and September 2020 and related the number of observed diagnosis cases to the expected number of cases (26). Consistent with our findings, Piontek et al. found a clear decline in the weeks of the first shutdown 2020, which continued, to a lesser extent, in the following 2 months. Very few new cases were registered for week 15 (April 6–12) (55.1% of the expected number of cases). However, other influencing factors cannot be ruled out.

Naturally, the presented data should be interpreted with caution and cannot be used to establish a causal relationship between the decrease in the rate of tumor board presentations and the influence of the first wave of the COVID-19 pandemic.

Because of the relatively low number of cases in each group analyzed here, our findings should also be validated by national registry data during further waves of the COVID-19 pandemic.

Nevertheless, NCT/UCC kept working as efficiently as before during the first wave of the COVID-19 pandemic, providing high quality care to cancer patients. Although a decrease in tumor board presentation rate was observed, it was not statistically significant for most entities and was within expected variability based on the data collected from 2014 to 2019.

To the best of our knowledge, our presented data are the first results from a German CCC that outline the influence of the first wave of the COVID-19 pandemic and could, therefore, be helpful in shaping future adjustments in the medical sector over the course of the pandemic.

We expect that the influence of the second wave of the COVID-19 pandemic will be much greater than that during the first shutdown.

The reasons for this are likely the massively increased number of illnesses and deaths worldwide and the much higher occupancy of intensive care units than during the first shutdown (27, 28).

In the near future, it is critical that the oncological community finds practical ways to cope with the expected onslaught of postponed tumor treatments and operations. Additional capacity to address the backlog of diagnostics and therapy might have the potential to minimize deaths. Evidence-based prioritization of patient groups for whom a delay would result in the highest loss of life-years warrants consideration.

Decision models, such as the one presented by Hartmann et al. could be helpful in the future (22).

We, therefore, advocate restricting the treatment of oncological patients as little as possible during the further course of the pandemic. Targeted countermeasures should be taken to avoid disastrous effects on the survival of oncological patients, as the pandemic is still ongoing.

## Data Availability Statement

The original contributions presented in the study are included in the article/Supplementary Material, further inquiries can be directed to the corresponding author/s.

## Ethics Statement

Ethical review and approval was not required for the study on human participants in accordance with the local legislation and institutional requirements. Written informed consent from the participants' legal guardian/next of kin was not required to participate in this study in accordance with the national legislation and the institutional requirements.

## Author Contributions

JK, JF, JW, and AR conceptualized the study. JK, AR, AK, VV, and SH collected clinical data and contributed to data analysis. GF, MK, VP, and TW gave substantial clinical input. JF, JW, and TW supervised the project. GF and MK provided infrastructure and gave important scientific input. JK drafted the initial manuscript text. All the authors read and approved the final manuscript.

## Conflict of Interest

The authors declare that the research was conducted in the absence of any commercial or financial relationships that could be construed as a potential conflict of interest.

## Publisher's Note

All claims expressed in this article are solely those of the authors and do not necessarily represent those of their affiliated organizations, or those of the publisher, the editors and the reviewers. Any product that may be evaluated in this article, or claim that may be made by its manufacturer, is not guaranteed or endorsed by the publisher.
