# Supplementary material for: Influence of the First Wave of the COVID-19 Pandemic on Cancer Care in a German Comprehensive Cancer Center
Source: Front Public Health. 2021 Nov 23;9:750479. doi: 10.3389/fpubh.2021.750479 (PMC8650694; doi:10.3389/fpubh.2021.750479)
Supplement: Supplementary file 1 [file Data_Sheet_1.docx]

Supplementary Material

# Supplementary Tables

*Tab. 1 suppl.: Most frequent tumor entities presented at the CCC from 01/2014 -10/2020*

| **Tumor entity** | **N (%)** |
| --- | --- |
| **Rectum** | 2515 (15.7) |
| **Colon** | 2294 (14.3) |
| **Pancreas** | 2090 (13.1) |
| **Liver (HCC and intrahepatic bile duct)** | 1855 (11.6) |
| **Stomach (incl. oesophagogastric junction)** | 1713 (10.7) |
| **Esophagus** | 1451 (9.1) |
| **Biliary tract (gall bladder, extrahepatic bile duct, ampulla vateri)** | 726 (4.5) |
| **Carcinoma of unknown primary (CUP)** | 635 (4.0) |
| **Small Bowel** | 332 (2.1) |
| **Endocrine organs** | 243 (1.5) |
| **Others** | 2141 (13.4) |
| **Total** | 15995 (100) |

*Tab. 2 suppl:* *Average change in tumor board presentation rate per treatment intention and year 2014 – 2020 and 01-10/2019 (pre-COVID-19 pandemic) versus 01-10/2020* (during COVID-19-pandemic)

| **Treatment intention** | **Average change/year 2014 – 2020**  **(%, [95 % CI)])*** | **01-10/2019**  **N**  **(% within 2019)** | **01-10/2020**  **N**  **(% within 2020)** | **Change 2020 vs. 2019**  **N (% change of 2019, [95 % CI])** |
| --- | --- | --- | --- | --- |
| **curative** | + 3.9% [1.7; 6.1] | 1171 (53.2) | 1088 (51.0) | - 83 (- 7.1%, [- 14.4; 0.9]) |
| **palliative** | + 4.4% [2.2; 6.7] | 660 (30.0) | 661 (31.0) | + 1 (+ 0.2%, [- 10.1; 11.6]) |
| **Not yet decided** | + 10.6% [5.8;15.6] | 370 (16.8) | 386 (18.1) | + 16 (+ 4.3%, [- 9.5; 20.3]) |
| **Total** | ­- | 2201(100) | 2135 (100) | - 66 (- 3.0% [- 8.6; 3.0]) |

*^* =time series analysis with a linear trend^*

*Tab. 3 Suppl: Average change in tumor board presentation rate per tumor entity and treatment intention 01-10/2019 (pre-COVID-19 pandemic) versus 01-10/2020 (during COVID-19 pandemic)*

| **Tumor entity** | **Treatment intention** | **01-10/2019**  **N (% within 2019)** | **01-10/2020**  **N (% within 2020)** | ***Change 2020* vs*. 2019***  ***N (%, [95 % CI]*)** |
| --- | --- | --- | --- | --- |
| **Biliary tract (gall bladder, extrahepatic bile duct, ampulla vateri)** | Curative  Palliative  Not yet decided  Total | 52 (47.7)  43 (39.4)  15 (13.6)  110 (100) | 49 (53.3)  29 (31.5)  14 (15.2)  92 (100) | - 3 (- 5.8%, [-36.1; 39.0])  - 14 (- 32.6%, [- 35.9; 11.4])  0 (0)  - 17 (- 15.6%, [- 57.5; 8.2]) |
| **Esophagus** | Curative  Palliative  Not yet decided  Total | 145 (66.0)  48 (21.8)  27 (12.3)  220 (100) | 108 (55.7)  47 (24.2)  39 (20.1)  194 (100) | - 37 (- 25.5%, [- 41.8; - 4.4])  - 1 (- 2.1%, [- 34.4; 46.1])  + 12 (+ 44.4%, [- 11.7; 133.7])  - 26 (- 11.8%, [- 27.3; 7.0]) |
| **Colon** | Curative  Palliative  Not yet decided  Total | 206 (61.1)  65 (19.3)  66 (19.6)  337 (100) | 170 (55.7)  68 (22.3)  67 (22.0)  305 (100) | - 36 (- 17.5%, [- 32.6; 1.1])  - 3 (- 4.6%, [- 25.5; 46.7])  - 1 (- 1.5%, [- 27.7; 42.4])  - 32 (- 9.5%, [- 22.5; 5.7]) |
| **Pancreas** | Curative  Palliative  Not yet decided  Total | 142 (43.4)  136 (41.6)  49 (15.0)  327 (100) | 136 (45.3)  110 (36.6)  52 (17.3)  300 (100) | - 6 (- 4.2%, [- 24.3; 21.1])  - 26 (- 19.1%, [- 37; 4])  - 3 (- 6.1%, [- 28.1; 56.4])  - 27 (- 8.3%, [- 21.5; 7.3]) |
| **Rectum** | Curative  Palliative  Not yet decided  Total | 252 (67.6)  62 (16.6)  59 (15.8)  373 (100) | 225 (63.0)  72 (20.2)  60 (16.8)  357 (100) | - 27 (- 10.7%, [- 25.4; 6.9])  + 10 (+ 16.1%, [- 17.3; 62.7])  + 1 (+ 1.7%, [- 28.9; 45.4])  - 16 (- 4.3%, [- 17.2; 10.7]) |

*Tab. 4 suppl: Average change in tumor board presentation rate per tumor stage 2014 – 2020 and 01-10/2019 (pre-COVID-19 pandemic) versus 01-10/2020 (during COVID-19 pandemic)*

| **Tumor stage** | ***Average change/year 2014 – 2020***  ***(%,*** [***95 % CI)***])******* | **01-10/2019**  **N (% within 2019)** | **01-10/2020**  **N (% within 2020)** | ***Change 2020* vs*. 2019***  ***N (%, [95 % CI]*)*)*** |
| --- | --- | --- | --- | --- |
| **Primary tumor** | + 6.2% [2.6; 10.1] | 1024 (45.9) | 1004 (46.5) | - 20 (-2.0%, [- 10.1; 7.0]) |
| **Local recurrent tumor** | + 10.4% [3.7; 17.5] | 171 (7.7) | 158 (7.3) | - 13 (-7.6%, [- 25.5; 14.7]) |
| **Metastases** | + 4,7% [1.5; 8.1] | 1037 (46.5) | 998 (46.2) | - 39 (-3.8%, [- 11.8; 5.0]) |
| **Total** | - | 2232 (100) | 2160 (100) | - 72 (- 3.2%, [- 8.8; 2.7]) |

*^* =time series analysis with a linear trend^*

*Tab. 5 suppl.:  Average change in tumor board presentation rate per tumor entity and tumor stage 01-10/2019 (pre-COVID-19 pandemic) versus 01-10/2020 (during COVID-19 pandemic)*

| **Tumor entity** | **Tumor stage** | **01-10/2019**  **N (% within 2019)** | **01-10/2020**  **N (% within 2020)** | ***Change 2020* vs*. 2019***  ***N (%, [95 % CI])*** |
| --- | --- | --- | --- | --- |
| **Biliary tract (gall bladder, extrahepatic bile duct, ampulla vateri)** | Primary tumor  Local recurrent tumor  Metastases  Total | 55 (50.0)  7 (6.4)  48 (43.6)  110 (100) | 52 (56.5)  1 (1.1)  39 (42.4)  92 (100) | + 3 (+ 5.5%, [- 35.1; 38.0])  - 6 (- 85.7%, [- 96.5; 15.4])  - 9 (- 18.7%, [- 46.5; 24.0])  -18 (- 16.4%, [- 36.5; 10.3]) |
| **Esophagus** | Primary tumor  Local recurrent tumor  Metastases  Total | 117 (53.2)  14 (6.4)  89 (40.5)  220 (100) | 101 (52.1)  18 (9.3)  75 (38.7)  194 (100) | - 16 (- 13.7%, [- 33.8; 12.7])  + 4 (+ 28.6%, [- 35.8; 153.7])  - 14 (- 15.7%, [- 37.9; 14.6])  - 26 (- 11.8%, [- 27.3; 7.0]) |
| **Colon** | Primary tumor  Local recurrent tumor  Metastases  Total | 110 (32.6)  10 (3.0)  217 (64.4)  337 (100) | 97 (31.8)  15 (4.9)  193 (63.3)  305 (100) | - 13 (- 11.8%, [- 32.8; 15.9])  + 5 (+ 50%, [- 32.6; 223.1])  - 24 (- 11.1%, [- 26.7; 8.0])  - 32 (- 9.5%, [- 22.5; 5.7]) |
| **Pancreas** | Primary tumor  Local recurrent tumor  Metastases  Total | 164 (50.2)  11 (3.4)  152 (46.5)  327 (100) | 158 (52.7)  12 (4.0)  130 (43.3)  300 (100) | - 6 (- 3.7%, [- 22.5; 19.8])  + 1(+ 9.1%, [- 51.2; 142.1])  - 22 (- 14.5%, [- 32.3; 8.1])  - 27 (- 8.3%, [- 21.5; 7.3]) |
| **Rectum** | Primary tumor  Local recurrent tumor  Metastases  Total | 117 (31.3)  38 (10.2)  219 (58.6)  374 (100) | 103 (28.8)  34 (9.5)  221 (61.7)  358 (100) | - 14 (- 12.0%, [- 32.4; 14.7])  -4 (- 10.5%, [- 43.4; 41.9])  + 2 (+ 0.9%, [- 16.3; 21.6])  - 16 (- 4.3%, [- 17.2; 10.6]) |
